# Research hotspots and trend analysis of cell transplantation in traumatic spinal cord injury: a bibliometric and visualized analysis

**DOI:** 10.3389/fphar.2023.1326583

**Published:** 2023-12-11

**Authors:** Yuhuai Guo, Bowen Gao, Shilin Sun, Jianuo Li, Xiaolin Lv, Hongna Yin, Zhongren Sun, Shuo Cai

**Affiliations:** ^1^ Department of TCM, Guangzhou Women and Children’s Medical Center of Guangzhou Medical University, Guangzhou, China; ^2^ The Second School of Clinical Medicine, Guangzhou University of Chinese Medicine, Guangzhou, China; ^3^ Heilongjiang University of Chinese Medicine, Harbin, China; ^4^ The Second Affiliated Hospital of Guangzhou Medical University, Guangzhou, China; ^5^ Department of Emergency, The Affiliated TCM Hospital of Guangzhou Medical University, Guangzhou, China

**Keywords:** cell transplantation, traumatic spinal cord injury, bibliometric analysis, marrow stromal cells, cell therapy, safety

## Abstract

**Background:** A traumatic spinal cord injury (TSCI) can lead to severe nerve damage and disability. Cell transplantation therapy has shown great potential in the reconstruction of damaged spinal cords and promoting functional recovery. However, there is a lack of frontiers and futures analysis in the study of cell transplantation in TSCI.

**Methods:** We used CiteSpace, VOSviewer and biblilometrix R package to perform bibliometric analysis on cell transplantation in TSCI from 2013 to 2023. Bibliometric records were extracted from English articles and reviews from the Web of Science core collection.

**Results:** The bibliometric analysis included 284 papers published in 154 journals by 1,780 authors from 487 institutions in 41 countries and regions. The number of articles published in the past decade has fluctuated slightly, while the number of article citations has steadily increased. Mainland China and the United States are the leading countries and regions in this field, with the National Natural Science Foundation of China being the most funded foundation, and the United States being the country with the most funded articles. The University of Toronto in Canada is a prolific institution. Michael G. Fehlings has published the most articles, and D Michele Basso is the most cited author. *Cell transplantation* is the most published journal, and *the Journal of Neurotrauma* is the most cited journal. Cell and tissue engineering and clinical neurology are the basic disciplines in this field, and cutting-edge disciplines include developmental biology, biochemistry and molecular biology, and materials science and multidisciplinary. This study also helps scholars understand the current hotspots and future trends in this field. Marrow stromal cells, glial progenitor, and cell therapy are current research hotspots in this field, while nerve regeneration, cell therapy, and the safety of transplantation of transplantation may be potential research directions in the future.

**Conclusion:** Cell transplantation after TSCI is receiving increasing attention. Cell therapy is both the frontier and a possible future trend in TSCI research. In addition, glial progenitor and marrow stromal cells are also current research hotspots. Meanwhile, nerve regeneration and safety of transplantation may be potential research directions. These findings will help further deepen research on cell transplantation for TSCI in scientific work.

## 1 Introduction

With the increase of global motor vehicles, aging population, and violent crime rate, the incidence of traumatic spinal cord injury (TSCI) has been increasing yearly (Chen et al., 2016; Jiang et al., 2022). TSCI is caused by primary mechanical injury (Ahuja et al., 2017). Unfortunately, secondary spinal cord injury can lead to more serious neurological damage and disability (Tran et al., 2018) and is currently one of the most serious public health problems (Ahuja et al., 2017). Hemodynamics, Methylprednisolone sodium succinate, decompressive surgery, and other methods are often used to treat TSCI, but complications such as neuropathic pain, neuropathic arthropathy, autonomic dysreflexia, genitourinary and gastrointestinal complications still occur (Ahuja et al., 2017). Therefore, it is necessary to explore effective treatment strategies to improve the quality of life of patients with TSCI.

Cell transplantation therapy has shown great potential in rebuilding damaged spinal cord and promoting functional recovery (Oliveri et al., 2014; Assinck et al., 2017). Schwann cells, neural stem cells, progenitor cells, mesenchymal stem cells, olfactory ensheathing cells, and precursor cells of oligodendrocytes are all considered potential cell transplantation options for the treatment of TSCI (Hu et al., 2010; Gómez et al., 2018; Ridler, 2018; Santamaría et al., 2018; Duncan et al., 2020). These cells often promote functional recovery after spinal cord injury by promoting neuronal regeneration, axon regeneration, myelin regeneration, immune regulation, and neuroprotection (Assinck et al., 2017).

Bibliometrics is a quantitative analysis of publications to identify leading countries, institutions, journals, authors, etc. in some fields, and to help researchers understand the current hotspots and future trends in this field (Bornmann and Leydesdorff, 2014; Rousseau, 2014; Ellegaard and Wallin, 2015). With the increasing attention on TSCI and rapid development of cell transplantation research, an increasing number of articles on cell transplantation for TSCI have been published in the past 10 years (Nori et al., 2011; Lu et al., 2012; Kadoya et al., 2016; Assinck et al., 2017; Levi et al., 2018; Cofano et al., 2019). Although there have been some bibliometric studies visualizing representative countries and journals in the field of spinal cord injury, there is a lack of discussion on cell transplantation (Kiraz and Demir, 2020). Additionally, there have been some systematic reviews discussing the research progress of neural stem cell therapy in preclinical and clinical settings of spinal cord injury, qualitatively exploring the contribution of neural stem cells to injury repair and functional recovery (Hosseini et al., 2023). However, quantitative analysis of a large body of literature is lacking (Hosseini et al., 2023). Furthermore, some review articles have analyzed the treatment strategies for spinal cord injury and future research directions, highlighting the need for objective research. But, these analyses remain qualitative rather than quantitative (Venkatesh et al., 2019). Therefor, bibliometric analysis of cell transplantation for TSCI research is still lacking. To fill this gap, we searched for studies on cell transplantation intervention for TSCI from 2013 to 2023, and conducted bibliometric analysis of these articles to sort out the core research forces in this field and clarify the trends and frontiers in this field.

## 2 Materials and methods

Publications on cell transplantation for TSCI from 2013 to 2023 were obtained from the Science Citation Index-Expanded dataset within the Web of Science Core Collection. Only review articles and original articles related to cell transplantation for TSCI, published in English, from 2013 to 2023, were included. Meanwhile, incomplete, duplicate, and irrelevant studies were excluded. The entire process of data retrieval and selection was independently performed by two authors, with any discrepancies resolved by a third reviewer.

**FIGURE 1:**
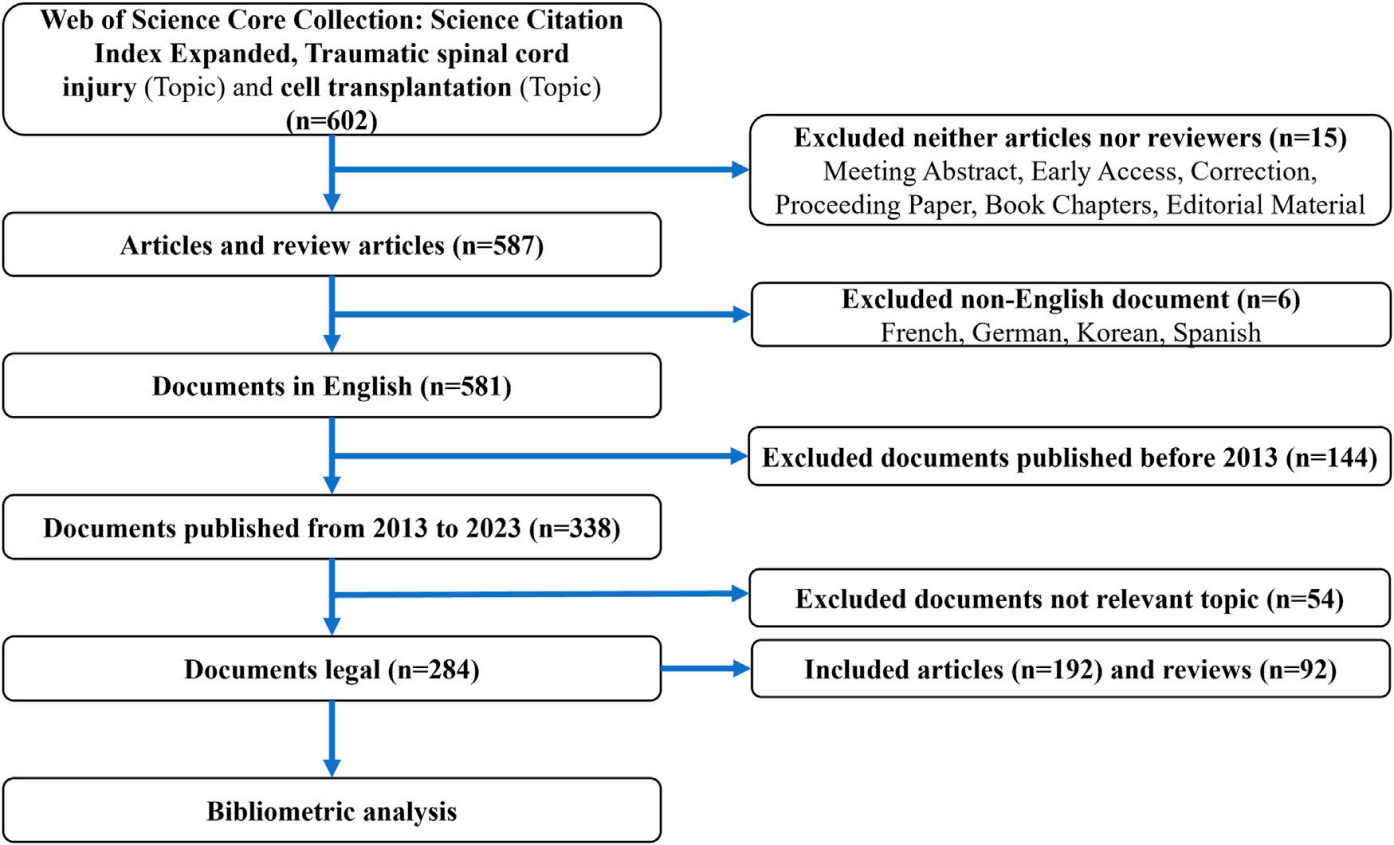
Flowchart of search strategy and bibliometric analysis.

CiteSpace 6.2.R4, Vosviewer 1.6.19 and Bibliometrix 4.1.2 are commonly used tools for bibliometric analysis and visualization. Each software has its advantages (Chen, 2006; van Eck and Waltman, 2017). Vosviewer and Bibliometrix was used to retrieve main information and visualize collaboration networks among countries, institutions, authors, and journals. CiteSpace was used for clustering of thematic categories, as well as visualizing networks, clusters, and bursts of keywords and references. In the generated network graphs, larger nodes indicate higher publication or citation counts, while thicker connections between nodes represent stronger associations. Furthermore, the colors of nodes and links signify different clusters (Liao et al., 2018; Donthu et al., 2021). Graphpad 9.5.1 was utilized to plot publication and citation trends, as well as to categorize thematic clusters (Figure 1).

## 3 Results

### 3.1 Main information

The time span of all the included data in this study is from 2013 to 2023. In the retrieved results, there were a total of 284 documents, including 192 articles and 92 reviews. On average, each document has been published for 4.7 years and has an average of 27.55 references cited. These documents were published in 154 different journals and had a total of 1,556 keywords, including 694 author keywords and 862 keywords plus. There were a total of 1,780 co-authors involved in these documents, with 3 authors publishing as sole authors. On average, each document had 7.17 authors, and international co-authorships accounted for 23.24%.

### 3.1 Publication and citation trends

**FIGURE 2:**
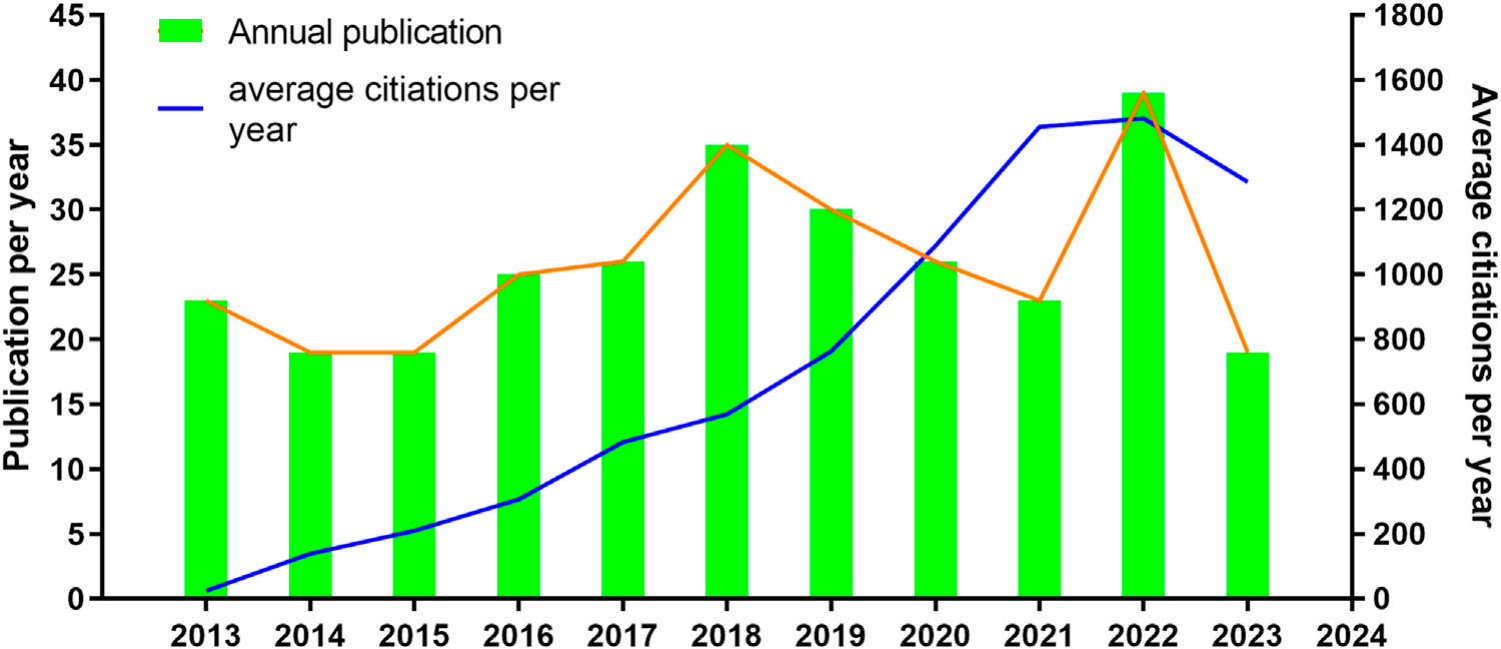
Publication and citation trends in the field of cell transplantation for TSCI.

In terms of the annual distribution of publications, the number of publications steadily increased from 2013 to 2018. However, there was a noticeable decrease in the number of publications from 2019 (n = 30) to 2021 (n = 23). In 2022, the number of publications rapidly rebounded and returned to the levels of 2018 and 2019 (Figure 2). Nevertheless, the citation count of the articles continued to increase annually until 2022, and it remained relatively stable in 2022, similar to the previous year. This suggests that the publication output experienced a slight slowdown from 2019 to 2021 due to certain objective factors, but the quality of articles in the field of cell transplantation for TSCI remains commendable Table 1.

**TABLE 1:** Main information about data.

Description	Results
Timespan	2013:2023
Sources (Journals, Books, etc.)	154
Documents	284
Document Average Age	4.7
Average citations per doc	27.55
References	15,195
Keywords Plus (ID)	862
Author’s Keywords (DE)	694
Authors	1780
Authors of single-authored docs	3
Co-Authors per Doc	7.17
International co-authorships %	23.24
article	192
review	92

### 3.2 Analysis of productive authors and highly cited authors

**FIGURE 3:**
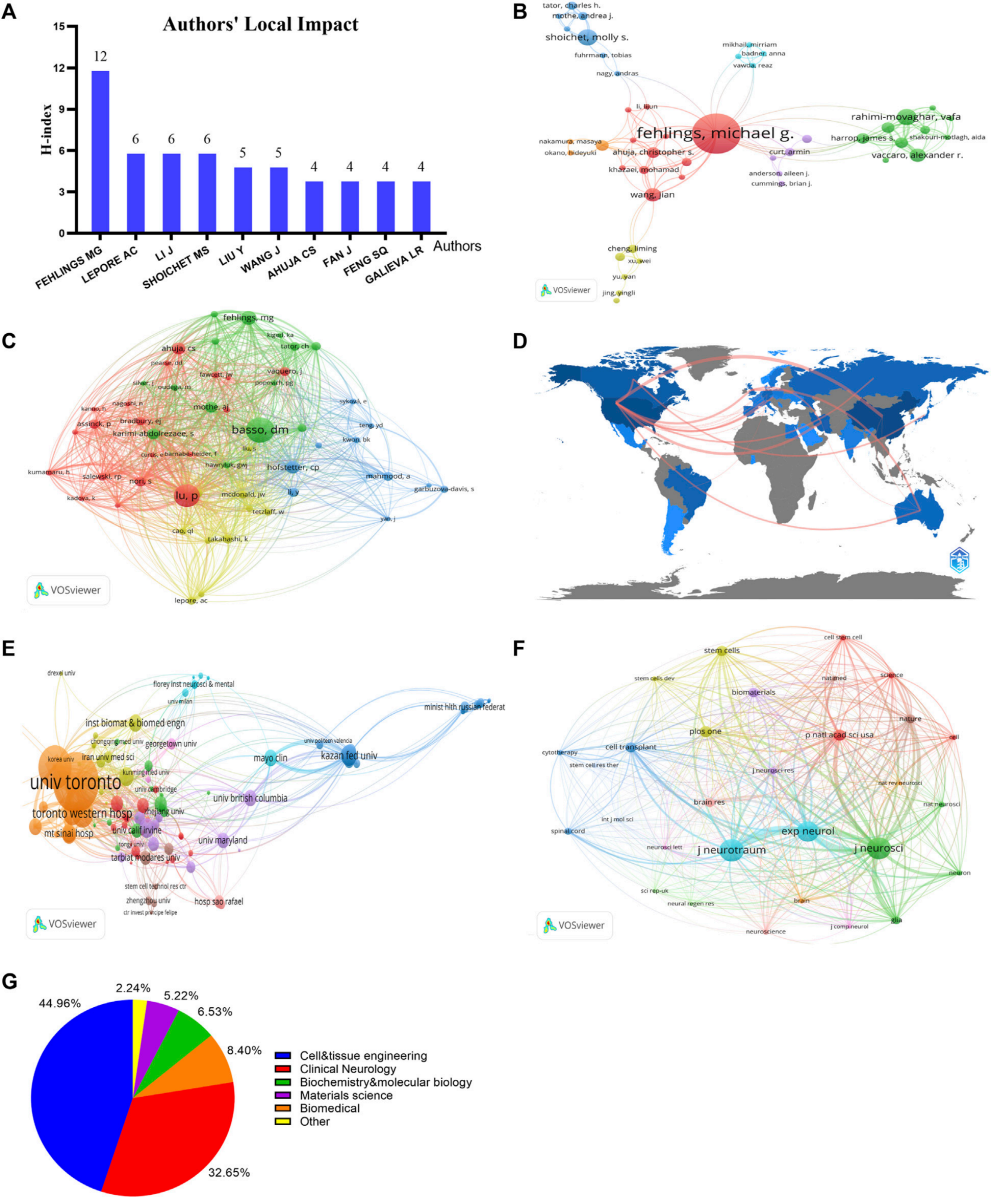
Distribution of Highly Cited Authors, Institutions, Countries, and Journals in the Field of cell transplantation for TSCI. **(A)** Local impact of authors; **(B)** Network of authors (n ≥ 2); **(C)** network of co-cited authors (n ≥ 5); **(D)** network map of countries (n ≥ 2); **(E)** network of institutions (n ≥ 2); **(F)** network of co-cited journals (top30); **(G)** distribution of disciplinary domains of publications.

A total of 1,780 authors have published articles in this field. Authors who contribute a large number of articles and authors who are highly cited both reflect their influence in a field. Michael G. Fehlings’s local impact is the highest (Figure 3A). Michael G. Fehlings, Vafa Rahimi-Movaghar and Molly S Shoichet are the top three authors with the highest number of publications (Table 2; Figure 3B). In terms of co-citation, D Michele Basso, Paul Lu, and Michael G. Fehlings are the most highly cited authors (Table 2; Figure 3C). It is evident that Michael G. Fehlings has the highest number of publications, approximately three times more than the second-ranked author, and is also among the top three in terms of co-citations. These findings indicate that Michael G. Fehlings is an important leader in the field of cell transplantation for TSCI.

**TABLE 2:** Prolific authors and highly cited authors.

Rank	Author	Counts	Author	Citations
1	Michael G. Fehlings	15	D Michele Basso	104
2	Vafa Rahimi-Movaghar	6	Paul Lu	94
3	Molly S Shoichet	6	Michael G. Fehlings	57
4	Alexander R Vaccaro	5	Christoph P Hofstetter	52
5	Wang Jian	5	Christopher S Ahuja	49
6	Christopher S Ahuja	4	Andrea J Mothe	46
7	James S Harrop	4	Soheila Karimi-Abdolrezaee	45
8	Mostafa Hosseini	4	Satoshi Nori	41
9	Narihito Nagoshi	4	Peggy Assinck	39
10	Mahmoud Yousefifard	4	Javier Vaquero	37

### 3.3 Analysis of countries and regions and funding

A total of 41 countries and regions have been found to contribute to cell transplantation for TSCI. The top three countries with the highest number of publications are PEOPLES R CHINA (n = 84), the United States (n = 82), and Canada (n = 26) (Figure 3D; Table 3). Some collaborations have been formed between different countries and regions, and the thicker lines between countries indicate closer collaborations, such as between China and the United States, German and Brazil, etc. (Figure 3D). In addition, the National Natural Science Foundation of China (n = 46), the National Institutes of Health (n = 28), and the United States Department of Health Human Services (n = 28) are the top three funding sources for publications. Among the top ten funding sources, those from the United States have funded the most articles.

**TABLE 3:** Leading countries/regions and funding.

Rank	Countries/Regions	Record count	Funding agencies	Record count	Countries/Regions
1	Peoples R China	84	National Natural Science Foundation of China	46	Peoples R China
2	United States	82	National Institutes of Health	28	United States
3	Canada	26	United States Department of Health Human Services	28	United States
4	Iran	19	Canadian Institutes of Health Research	15	Canada
5	Brazil	16	National Institute of Neurological Disorders Stroke	12	United States
6	Germany	15	Spanish Government	8	Spain
7	Russia	13	European Union	7	European Union
8	Australia	11	Krembil Foundation	7	Canada
9	England	11	Tehran University Of Medical Sciences	7	Iran
10	Spain	9	Uk Research Innovation	7	United Kingdom

### 3.4 Analysis of institutions and departments

A total of 487 institutions have published articles on cell transplantation for TSCI. The University of Toronto, with 23 publications, is the institution with the highest output in this field (Table 4). Next are the University Health Network and Krembil Research Institute. The departments with the highest number of publications are Toronto Western Hospital, University of Toronto Temerty Faculty of Medicine, and University of Toronto Department of Surgery (Table 4). It can be seen that Department of Surgery (13 in 23) and Temerty Faculty of Medicine (13 in 23) are the main contributor of the University of Toronto in this field. The collaboration network among institutions shows that the University of Toronto is the leading institution in the field of cell transplantation for TSCI (Figure 3E).

**TABLE 4:** Top institutions and departments.

Rank	Affiliations	Record count	Affiliation with department	Record count
1	University of Toronto	23	University of Toronto Department of Surgery	13
2	University Health Network Toronto	18	University of Toronto Temerty Faculty of Medicine	13
3	Krembil Research Institute	15	Sina Hospital	6
4	Jefferson University	12	Sina Trauma And Surgery Research Center	6
5	Tehran University of Medical Sciences	12	Kazan Federal University Institute of Fundamental Medicine And Biology	5
6	Kazan Federal University	8	Tehran University of Medical Sciences School of Medicine	5
7	Kazan State Medical University	8	Thomas Jefferson University Sidney Kimmel Medical College	5
8	University of California System	8	University Of Toronto Division Of Neurosurgery	5
9	Nanjing Medical University	7	Cairo University Faculty Of Veterinary Medicine	4
10	Tianjin Medical University	7	Tehran University of Medical Sciences School of Medicine	4

### 3.5 Analysis of journals with the most publications and highest citation counts

From 2013 to 2023, a total of 154 journals have accepted articles in the field of cell transplantation for TSCI. cell transplantation (n = 12) is the journal with the highest number of publications in this field, followed by Stem Cell Research Therapy (n = 10) and Neural Regeneration Research (n = 9) (Table 5; Figure 3F). In addition, Journal of Neurotrauma (n = 837) is the journal with the highest citation count, followed by Journal of Neuroscience (n = 811) and Experimental Neurology (n = 753).

**TABLE 5:** Top prolificand and co-cited journals.

Rank	Publication titles	Record count	Co-cited source	Citations
1	Cell Transplantation	12	Journal of Neurotrauma	837
2	Stem Cell Research & Therapy	10	Journal of Neuroscience	811
3	Neural Regeneration Research	9	Experimental Neurology	753
4	Frontiers In Cellular Neuroscience	7	PNAS	416
5	Journal Of Neurotrauma	7	Plos One	416
6	Cells	6	Cell Transplant	377
7	Cytotherapy	6	Stem Cells	373
8	Stem Cells Translational Medicine	6	Biomaterials	348
9	Plos One	5	Brain Research	341
10	Biomaterials	4	Nature	332

### 3.6 Distribution of disciplinary domains of publications

Through CiteSpace’s co-occurrence analysis of disciplinary categories, it was found that there are 8 clusters, primarily including Cell and Tissue Engineering (44.96%), Clinical Neurology (32.65%), and Biomedical (8.40%). These three disciplines have been the most popular from 2013 to 2023 (Figure 3G). In terms of centrality and average age, the most prominent disciplinary classifications are Biochemistry and Molecular Biology (1.0, 2018), Pathology (1.0, 2017), and Materials Science and Multidisciplinary (0.976, 2017). Clearly, the current research on cell transplantation for traumatic spinal cord injuries is an interdisciplinary field that combines Cell and Tissue Engineering and Clinical Neurology, incorporating disciplines such as Biochemistry, Molecular Biology, and Developmental Biology.

### 3.7 Cluster of highest frequency keywords and top bursting keywords

**FIGURE 4:**
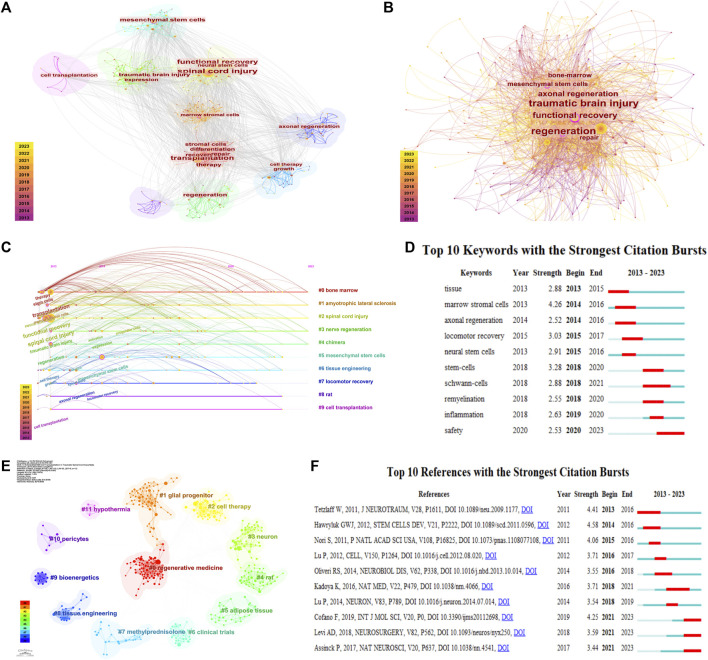
Cluster, frequency and burst of keywords and reference. **(A)** frequency of keywords; **(B)** centrality of keywords; **(C)** timeline and clustering of keywords; **(D)** bursting keywords; **(E)** clustering of reference; **(F)** bursting reference.

In addition to spinal cord injury and transplantation, the highest frequency keywords are functional recovery, mesenchymal stem cell, and regeneration (Figure 4A). Traumatic brain injury (0.16), regeneration (0.16), function recovery (0.14), axonal regeneration (0.13) and mesenchymal stem cell (0.11) are keywords with centrality above 0.1, occupying central positions in the keyword network (Figure 4B). A total of 10 clusters were identified through keyword clustering, with bone marrow (n = 56), amyotrophic lateral sclerosis (n = 46), and spinal cord injury (n = 32) being the largest clusters. The average publication year for Cluster #0 bone marrow, Cluster #3 nerve regeneration, and Cluster #4 chimera is 2017, indicating the cutting-edge research in cell transplantation for TSCI (Figure 4C). Bursting keywords reflect the temporal hotspots. Among the top 10 bursting keywords, marrow stromal cells (4.26) exhibit the strongest burst, while the latest topics in safety (2020–2023) suggest future research trends (Figure 4D).

### 3.8 Co-citation analysis of references

Co-cited references are the references that are commonly cited together in a group of publications. The references of 284 documents included in this analysis can be divided into 12 clusters. The largest clusters are related to regenerative medicine, glial progenitor, and cell therapy (Figure 4E). The clusters with the most recent average publication years are neuron (2019), cell therapy (2019), and pericytes (2016). Assinck et al., 2017 has the highest number of citations (n = 27) (Assinck et al., 2017), while (Anderson et al., 2017) has the highest centrality score (0.29) (Anderson et al., 2017) (Table 6). The reference with the highest burst intensity is (Hawryluk et al., 2012) (4.58) (Cofano et al., 2019; Cofano et al., 2019; Assinck et al., 2017; Levi et al., 2018) are the most recent bursting references, providing some insights into predicting future research trends (Figure 4F) (Assinck et al., 2017; Levi et al., 2018; Cofano et al., 2019).

**TABLE 6:** Top10 Co-cited reference.

Title	First author	Journal	Impact factor	Year	Citations (DOI)	Research Category ranking	Counts	Centrality
Cell transplantation therapy for spinal cord injury	Assinck P	Nature Neuroscience	25	2017	10.1038/nn.4541	JCR Q1	27	0.24
Safety of Autologous Human Schwann Cell Transplantation in Subacute Thoracic Spinal Cord Injury	Anderson KD	Journal of Neurotrauma	4.2	2017	10.1089/neu.2016.4895	JCR Q2	18	0.29
A First-in-Human, Phase I Study of Neural Stem Cell Transplantation for Chronic Spinal Cord Injury	Curtis E	Cell Stem Cell	23.9	2018	10.1016/j.stem.2018.05.014	JCR Q1	18	0.01
Spinal cord reconstitution with homologous neural grafts enables robust corticospinal regeneration	Kadoya K	Nature Medicine	82.9	2016	10.1038/nm.4066	JCR Q1	17	0.28
Traumatic spinal cord injury	Ahuja CS	Nature Reviews Disease Primers	81.5	2017	10.1038/nrdp. 2017.18	JCR Q1	16	0.04
Restorative effects of human neural stem cell grafts on the primate spinal cord	Rosenzweig ES	Nature Medicine	82.9	2018	10.1038/nm.4502	JCR Q1	15	0.06
Mesenchymal Stem Cells for Spinal Cord Injury	Cofano F	International Journal of Molecular Sciences	5.6	2019	10.3390/ijms20112698	JCR Q1	13	0.02
Emerging Safety of Intramedullary Transplantation of Human Neural Stem Cells in Chronic Cervical and Thoracic Spinal Cord Injury	Levi AD	Neurosurgery	4.8	2018	10.1093/neuros/nyx250	JCR Q1	11	0.07
Traumatic Spinal Cord Injury-Repair and Regeneration	Ahuja CS	Neurosurgery	4.8	2017	10.1093/neuros/nyw080	JCR Q1	11	0.03

## 4 Discussion

### 4.1 Analysis of leading countries, funds, institutions, journals, and authors

In this study, we conducted a bibliometric analysis of scientific achievements in the field of cell transplantation for TSCI published from 2013 to 2023 using CiteSpace and VOSviewer. A total of 284 papers were included, authored by 1780 authors from 487 institutions across 41 countries and regions and published in 154 journals. As for the trend of publications and citations from 2013 to 2023, the number of publications showed a steady growth trend with a possible decrease in 2019–2021 due to certain reasons, while the overall citation count kept increasing. It is worth noting that the decline in publication volume during 2019–2021 may be related to the COVID-19 pandemic (Arora et al., 2021; Shamseer et al., 2021). However, the similar local citation count in 2022 compared to 2021 could be due to the fact that only publications up to 24 November 2023 were counted. In addition, considering that publications from 2022 may only be cited by other publications from 2022 to 2023 so far, the average citation count in 2022 may not have actually decreased. These findings indicate that despite some fluctuations, the publications and citation counts in this field have been on the rise in the past decade and their quality is recognized.

Cell transplantation for TSCI is a multidisciplinary research field. After clustering the disciplinary categories of 284 literature using CiteSpace, we found that cell and tissue engineering, clinical neurology, and materials science, multidisciplinary were the top three popular disciplines in the past decade. This further illustrates that the current research focus in this field is primarily rooted in cell and tissue engineering (44.96%) and clinical neurology (32.65%). Though developmental biology (0.963, 2018), biochemistry and molecular biology (1.0, 2018), and materials science and multidisciplinary studies (0.975, 2017) might not have the highest number of projects in the clustering analysis, they still exhibit strong centrality and relatively recent average publication years, indicating their prominence as popular disciplinary categories for current and future research. These findings highlight that the research field of cell transplantation for TSCI is based on the foundation of cell and tissue engineering and clinical neurology, incorporating interdisciplinary realms such as developmental biology, biochemistry and molecular biology, and materials science and multidisciplinary studies.

Cell transplantation, Stem Cell Research Therapy, and Neural Regeneration Research are the most prolific journals in this field and are considered authoritative. Journal of Neurotrauma, Journal of Neuroscience, and Experimental Neurology are the most highly cited journals in this domain.

China, with 84 articles published, the United States, with 82 articles, and Canada, with 26 articles, have made the greatest contributions to this field. Among them, the National Natural Science Foundation of China is the most funded foundation for these articles, while the United States is the country with the most funded articles in this area. Interestingly, the University of Toronto (23/26), University Health Network (18/26), and Krembil Research Institute (15/26) from Canada are the institutions that have published the most articles globally and are the absolute protagonists of Canada in this field. Interestingly, Temerty Faculty of Medicine (13/23), and Department of Surgery (13/23) from the University of Toronto are not only the departments that have published the most articles globally in this field but also the major contributors in Canada, indicating that cooperation between institutions in Canada is the most active.

Michael G. Fehlings is the author who has published the most articles in the field of cell transplantation for TSCI, with 15 documents far exceeding the second-ranked author Vafa Rahimi Movaghar’s 6 documents. Interestingly, he is also one of the top three authors with the most citations. D Michele Basso and Paul Lu have been cited in almost 100 articles, which means that almost one-third of the articles have cited their works (104/284, 94/284). Overall, Michael G. Fehlings, D Michele Basso, Paul Lu are the leading figures in this field.

### 4.2 Hotspots and trends

In addition to the main keywords “spinal cord injury” and “cell transplant,” the most frequent keywords include “functional recovery,” “mesenchymal stem cell,” and “regeneration.” This indicates that the primary aim of cell transplantation for TSCI is functional recovery and regeneration, and mesenchymal stem cell serve as the main approach for cell transplantation.

The largest keyword cluster in this field is “bone marrow” and “amyotrophic lateral sclerosis,” while “marrow stromal cells” is the most explosive keyword. This suggests that they are still the hot topics of current research in this field. “bone marrow,” “nerve regeneration” and “chimera” represent the latest cluster, and “safety” (2021–2023) is the most recent explosive keyword, indicating a potential trend for ongoing research in the future. It can be observed that marrow stromal cells are an important type of transplanted cells for TSCI, while nerve regeneration and safety represent a future research trend in this field.

Additionally, we note that regeneration medicine, glial progenitor and cell therapy are the largest cluster, and neuron, cell therapy and pericytes are the most recent reference cluster. This indicates that cell therapy will continue to be a significant topic of interest in the field of cell transplantation for TSCI.

(Assinck et al., 2017; Levi et al., 2018; Cofano et al., 2019) are the most recent high-impact reference articles (Assinck et al., 2017; Levi et al., 2018; Cofano et al., 2019). Interestingly, (Cofano et al., 2019), (4.25) is not only the most recent high-impact reference article, but also the one with the highest burst intensity. This article mainly reviews the features, applications, limitations and future prospects of mesenchymal stem cell transplantation for spinal cord injury (Cofano et al., 2019; Assinck et al., 2017), on the other hand, is not only the most recent high-impact reference article, but also the most cited one. It provides a detailed introduction to the cell types commonly used for transplantation to treat spinal cord injury, as well as the mechanisms by which these cell transplants promote functional recovery (Assinck et al., 2017; Levi et al., 2018)’s article mainly discusses the increasing safety of intramedullary transplantation of human neural stem cells for cervical and thoracic spinal cord injury (Levi et al., 2018). Coincidentally, (Anderson et al., 2017), (0.29) is the most central reference article, which suggests that it is feasible to obtain autologous stem cells from peripheral nerves within 5–30 days after acute spinal cord injury and perform highly purified autologous stem cell transplantation within 4–7 weeks after injury. This proves that autologous hematopoietic stem cell transplantation for subacute thoracic spinal cord injury is safe (Anderson et al., 2017). It should be noted that safety is also the most recent explosive keyword. Considering this, we predict that safety of cell transplantation will also be a major focus in future research.

### 4.3 Advantages and limitations

There are still some inevitable limitations in this study. Firstly, using Web of Science as the only retrieval database may result in some legitimate publications being missed, even though other databases were considered for analysis with a wide range of data available (Zhu and Liu, 2020). Secondly, the credibility of the articles may be affected by the relatively small number of literature included in this study, despite the increasing number of studies on cell transplantation for TSCI (Feng et al., 2021). Lastly, the development trends and topics can be influenced by various biases such as publication bias and citation bias (Urlings et al., 2021), as well as biases based on different algorithms of VOSviewer and CiteSpace software frameworks (Ding and Yang, 2022).

Fortunately, meaningful results in the field of cell transplantation for TSCI have been identified. Firstly, this study provides a framework of research output in the field of TSCI since 2013, which can assist interested scholars in their search for relevant information. In this field, Mainland China and the United States are leading countries and regions, with the National Natural Science Foundation of China being the most frequently funded foundation for the included articles and the United States being the country with the highest number of articles funded by foundations. The University of Toronto in Canada is the institution with the highest number of published articles. Michael G. Fehlings is the author with the highest number of published articles, and Dm Basso is the co-author with the highest number of citations. Cell transplantation is the journal with the highest number of publications, while the Journal of Neurotrauma is the journal with the highest number of citations. Cell and tissue engineering and clinical neurology are the foundational disciplines in this field, integrating frontier disciplines such as developmental biology, biochemistry and molecular biology, and materials science and multidisciplinary. Secondly, this study helps researchers understand the current hotspots and future trends in this field. Marrow stromal cells, glial progenitor, and cell therapy are the current hot topics in this field. Nerve regeneration, cell therapy, and the safety of transplantation may be the future research trends.

## 5 Conclusion

Cell transplantation after TSCI is receiving increasing attention. Cell therapy is both at the forefront of traumatic spinal cord research and a potential research trend for the future. Additionally, glial progenitor and marrow stromal cells remain hot topics in current research. Furthermore, nerve regeneration and safety of transplantation are potential research directions. These findings contribute to further in-depth research on cell transplantation for TSCI, aiding scientific work in this field.

## Data Availability

This article/Supplementary Material contains the original contributions presented in the study. Further inquiries can be directed to the corresponding authors.
